# Implementation of a Regional Training Program on African Swine Fever As Part of the Cooperative Biological Engagement Program across the Caucasus Region

**DOI:** 10.3389/fvets.2017.00164

**Published:** 2017-10-26

**Authors:** Marco De Nardi, Anaïs Léger, Tatul Stepanyan, Bagrat Khachatryan, Talgat Karibayev, Igor Sytnik, Samat Tyulegenov, Assel Akhmetova, Serhiy Nychyk, Mykola Sytiuk, Oleg Nevolko, Roman Datsenko, Tengiz Chaligava, Lasha Avaliani, Otar Parkadze, Lena Ninidze, Natia Kartskhia, Tsira Napetvaridze, Zviad Asanishvili, Demna Khelaia, Ioseb Menteshashvili, Meruzhan Zadayan, Lyudmila Niazyan, Nataliya Mykhaylovska, Bradford Raymond Brooks, Gulnara Zhumabayeva, Saltanat Satabayeva, Magda Metreveli, Theresa Gallagher, Richard Obiso

**Affiliations:** ^1^SAFOSO, Liebefeld, Switzerland; ^2^Veterinary and Animal Husbandry Department, Ministry of Agriculture, Yerevan, Armenia; ^3^Republican Veterinary Sanitary and Phyto-Sanitary Center of Laboratory Services, State Service for Food Service, Ministry of Agriculture, Yerevan, Armenia; ^4^National Reference Center for Veterinary Medicine, Ministry of Agriculture, Astana, Kazakhstan; ^5^Institute of Veterinary Medicine, Kyiv, Ukraine; ^6^State Scientific Research Institute of Laboratory Diagnostics and Veterinary Sanitary Expertise, Kyiv, Ukraine; ^7^National Food Agency of the Ministry of Agriculture, Tbilisi, Georgia; ^8^CH2M HILL Reginoal Office, Yerevan, Armenia; ^9^Metabiota Regional Office, Kyiv, Ukraine; ^10^AECOM Regional Office, Almaty, Kazakhstan; ^11^CH2M HILL Regional Office, Tbilisi, Georgia; ^12^Avila Scientific, Christiansburg, VA, United States

**Keywords:** Cooperative Biological Engagement Program, African swine fever virus, Armenia, Georgia, Kazakhstan, Ukraine, Public Outreach, pedagogy

## Abstract

A training and outreach program to increase public awareness of African swine fever (ASF) was implemented by Defense Threat Reduction Agency and the Ministries of Agriculture in Armenia, Georgia, Kazakhstan, and Ukraine. The implementing agency was the company SAFOSO (Switzerland). Integration of this regional effort was administered by subject matter experts for each country. The main teaching effort of this project was to develop a comprehensive regional public outreach campaign through a network of expertise and knowledge for the control and prevention of ASF in four neighboring countries that experience similar issues with this disease. Gaps in disease knowledge, legislation, and outbreak preparedness in each country were all addressed. Because ASF is a pathogen with bioterrorism potential and of great veterinary health importance that is responsible for major economic instability, the project team developed public outreach programs to train veterinarians in the partner countries to accurately and rapidly identify ASF activity and report it to international veterinary health agencies. The project implementers facilitated four regional meetings to develop this outreach program, which was later disseminated in each partner country. Partner country participants were trained as trainers to implement the outreach program in their respective countries. In this paper, we describe the development, execution, and evaluation of the ASF training and outreach program that reached more than 13,000 veterinarians, farmers, and hunters in the partner countries. Additionally, more than 120,000 booklets, flyers, leaflets, guidelines, and posters were distributed during the outreach campaign. Pre- and post-ASF knowledge exams were developed. The overall success of the project was demonstrated in that the principles of developing and conducting a public outreach program were established, and these foundational teachings can be applied within a single country or expanded regionally to disseminate disease information across borders; overall, this method can be modified to raise awareness about many other diseases.

## Background and Rationale for the Educational Activity

Introduction of African swine fever virus (ASFV) into domestic pig populations can incite economic catastrophe, resulting in loss of agricultural assets with an adverse impact on the international commercial pork trade (1).

African swine fever (ASF) is a highly infectious disease of most species in the Suidae family, including domestic pigs and African and European wild pigs (however, warthogs and bush-pigs are unaffected). ASF is a vector-borne viral disease caused by an asfivirus, a DNA virus in the *Asfarviridae* family. Infection with this virus can result in hemorrhagic fever, causing high morbidity and mortality. The virus is efficiently transmitted from wild boars to domestic pigs, and between live pigs through contact or ingestion of contaminated meat. Soft ticks (*Ornithodoros* species) are the only known vectors of ASF (1). The presence, distribution, and epidemiological role of *Ornithodoros* ticks in the Caucasus region and eastern Europe remains unclear (2).

Highly lethal and easily spread among domestic pig populations, ASF is one of the most serious transboundary swine diseases, often leading to significant socioeconomic consequences in countries where outbreaks occur. Before 1957, ASF had been detected only in Africa (1). The first outbreak of ASF was recorded in Georgia in April 2007; ASF cases were simultaneously reported in three different locations across the country. Soon after the outbreak in Georgia, ASF was detected in the northeast regions of Armenia; in 2010 and 2011, new outbreaks were detected in other regions of the country. ASF was reported in Ukraine in 2012 and has spread across different oblasts (counties within a country) and independent republic regions north of the Caucasus including Russia’s Orenburg oblast (near the border with Kazakhstan), and the Ingushetia Republic and Stavropol Krai (near the Georgian border) (3).

This paper describes the implementation and evaluation of a public outreach campaign designed to bring together countries with shared regional interests to collaboratively develop a training program to increase public awareness about ASF and mitigate disease risk and economic losses.

## Rationale

The training program was first developed in 2015 through a collaborative effort between the US Defense Threat Reduction Agency and the governments of Armenia, Georgia, Kazakhstan, and Ukraine. These four countries were selected for participation because of the importance of the pork industry as an agricultural asset and their potential for future ASF outbreaks. These four countries are located in the same general region and experience similar issues with ASF. The Ministries of Agriculture from each partner country implemented this project, with mentoring and support from SAFOSO, Biological Threat Reduction Integrating Contractors (BTRICs), and subject matter experts (SMEs). Important notes about the methodology followed were kept by each country so that similar outreach campaigns could be adapted to other diseases.

## Pedagogical Framework

The pedagogical framework creates the structure around the philosophy of teaching and learning. In this project, the project team mentors developed guidelines that outlined teaching and training best practices to share with representatives of the partner countries. The goal was to establish cross-border cooperation and to identify and introduce training methodologies that could be implemented across similar but divergent cultures for a common disease. In this regard, the hope was that all veterinarians who were trained (the students) could reach their full potential in sharing information on ASF with relevant audiences in their home countries. The main framework was to provide the trainers from each of these partner countries with the ability to create and deliver jointly developed curriculum based on best practices (as described by the facilitators and SMEs) and then relate it to the most effective way that students learn in their home countries. The goal of this project was to use a training-of-trainers (ToT) approach to enhance the regional network for ASF control and prevention by increasing awareness among workers who directly handle swine, and among governmental organizations responsible for preventing and responding to outbreaks in the Caucasus. Ultimately, this project has helped to establish a general approach for conducting and deploying a public outreach program. The fundamental principles of how this program was developed and implemented not only helped raise awareness about ASF but can also be applied to help mitigate future outbreaks of other diseases. Most importantly, this project also served as a basis for developing much-needed public outreach programs for other diseases of public concern.

## Learning Objectives

**Figure 1:**
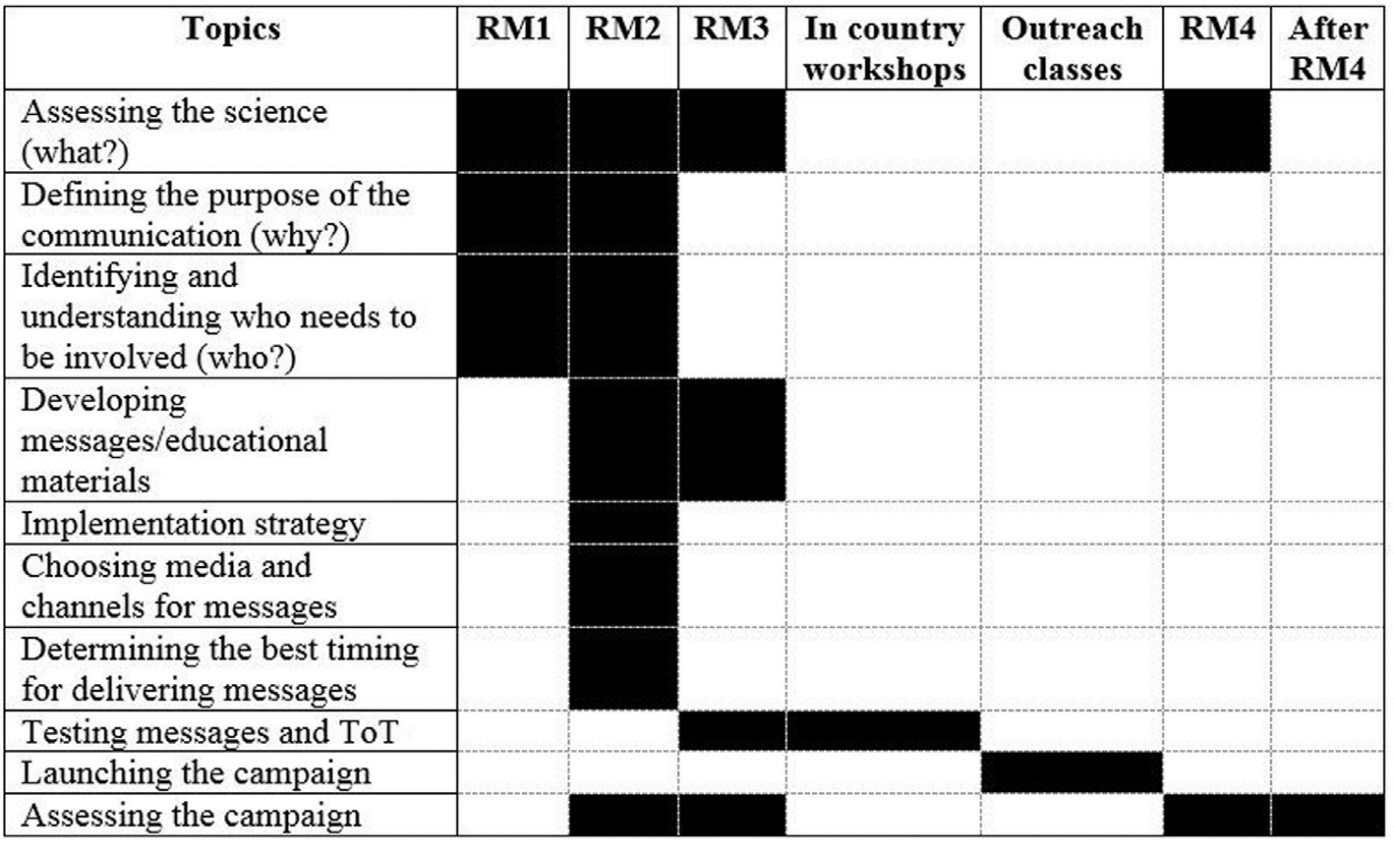
Topics covered during project development meetings and training activities for developing the Public Outreach Campaign. RM1 refers to the first regional meeting (RM) that took place in Armenia between 03 and 05 February 2015. RM2 was the second RM in Kazakhstan between 23 and 27 March 2015. RM3 was the third RM in Ukraine between 18 and 22 May 2015. RM4 was the final RM that took place in Georgia between 03 and 04 November 2015. These activities outline the development and implementation of the Public Outreach campaign.

The project adapted a communication plan that was developed using the framework of the Canine Rabies Blueprint (http://caninerabiesblueprint.org/Communications-plan?lang=en), to serve as a guideline for raising awareness about diseases. The 10 steps were the basis for project objectives and activities and were addressed during different project events [regional meetings (RMs), in-country independent workshops, and in-country outreach classes]. Most tasks and topics covered in the project development meetings and training activities are shown in Figure 1, and were completed over the course of more than one joint meeting of the trainers.

The activities outlined in Figure 1 formed the basis for the tasks that expanded the development and implementation of the Public Outreach campaign. From a methodological point of view, a collaborative effort was required from country delegates across the four representative countries who actively participated in working group sessions in each of the RMs. The outputs from these highly interactive sessions formed the foundation for the entire outreach program.

## Training Competencies and Standards Underlying the Educational Activity

The underlying purpose of this project was to identify persons working in the pig production chain (pig keepers, butchers, middlemen, hunters, farmers, etc.) and teach them how to recognize clinical signs and epidemiological patterns of ASF. Specifically, this included (i) common routes of exposure, (ii) preventative measures, (iii) how to recognize clinical signs and the importance of reporting to veterinary authorities, and (iv) how to respond to suspected ASF cases.

To leverage these efforts in support of risk mitigation throughout regions of Eastern Europe that serve as focal points for ASF transmission, a regional approach was used to engage agricultural leadership (Ministries of Agriculture) from Armenia, Georgia, Kazakhstan, and Ukraine in collaboratively developing strategies for enhancing ASF public awareness in their respective countries.

To strengthen the regional approach, this group oversaw the development and implementation of these learning objectives to eventually administer this training program in their home countries. Ultimately, we hoped to reduce the risk of epizootic emergence and economic losses from ASFV infection by formally educating those who can help to prevent its spread, including farmers, hunters, wild fauna management staff, and people engaged in the commercial pork supply chain. A key part of the training program was to stress the importance of reporting suspected outbreaks to the veterinary authorities and neighboring communities so that the disease and subsequent outbreaks can be swiftly controlled. An early reaction is critical to minimizing the impact of an ASF outbreak.

This project’s overarching goal, and, collateral benefit, however, was to foster fellowship between project participants through achievement of a common goal that was based on shared interests, concerns, and challenges, providing a foundation upon which to build the capacity for future collaborative pursuits.

## Learning Environment

### Setting

To bring together four neighboring countries to develop and implement a public outreach campaign, the facilitators and SMEs set up a series of RMs, workshops, and outreach classes (Table 1) in Armenia, Georgia, Kazakhstan, and Ukraine throughout 2015. Table 1 summarizes and outlines the activities that were completed during development and implementation of the Public Outreach campaign. RM1 refers to the first RM that took place in Armenia. RM2 was the second RM in Kazakhstan. RM3 was the third RM in Ukraine. RM4 was the final RM that took place in Georgia. Implementation of the training workshops and the outreach program were conducted between RM3 and RM4.

**Table 1:** Objectives, timing, and location of regional meetings (RMs), workshops, and development of the overall training program for the implementation of the public outreach project.

Event	Month (2015)	Location	Objective
RM # 1	February	Yerevan, Armenia	Determine the scope of the outreach programs
RM # 2	March	Almaty, Kazakhstan	Plan and develop the outreach content including messages and materials
RM # 3	May	L’viv, Ukraine	Establish capacity (technical and didactical) to train the trainers
In-country workshops	June to August	Country-specific workshops	Train the trainers in each country
Country outreach classes	August to October	Country-specific classes	Enhance target group awareness of African swine fever
RM # 4	November	Tbilisi, Georgia	Evaluate performance, successes, and difficulties of training activities

Each RM and workshop encompassed specific objectives, and the organization of working group sessions brought together partner country participants to address a common disease and shared public outreach effort. During the working group sessions, country-specific educational materials, implementation strategies, and methodologies were developed. In one of the first working group sessions, participants conducted gap analyses to identify limitations in the educational activities and materials that had been previously deployed in their home countries. Through this analysis, participants identified the best approaches to use in outreach and pitfalls from past programs to avoid.

### Development of Training Materials

During the second RM, country representatives shared previously developed presentations on ASF that would later form the bases of their country-specific presentations. The purpose of this session was two-fold: first, to introduce participants to the latest information about ASF, and, second, to provide feedback on their individual country-specific presentations so that presenters could refine them before delivery to the target audiences. ASF was discussed in detail, including its etiology, epidemiology, transmission, pathogenesis and immunology, clinical signs and diagnostics, and mechanisms of prevention and control. Country representatives presented relevant ASF educational and awareness materials (e.g., leaflets, brochures, training manuals, radio/TV broadcast messages, and contingency plans) that were already available in their countries. These existing materials were used to guide working groups as they selected content for new educational materials to be developed. Participants in the working groups were asked to (i) revise existing educational materials; (ii) select relevant materials to be used in the outreach programs; (iii) develop a specific communication toolkit for each target group; (iv) identify gaps and areas for improvement in the selected materials; (v) choose the key messages of the outreach program; and (vi) update and develop educational materials to include in the communication toolkit. Participants included the latest scientific information in the materials whenever possible. Finally, training materials were compiled and printed for distribution to target groups. Each country targeted different groups in their communication toolkits. Armenia’s communication toolkit is shown in Table 2 as an example. A communication toolkit targeting large farms and places that are frequently visited by people related to the pig production industry was developed in Kazakhstan. This included the distribution of 176 posters and 7,290 leaflets to farmers and hunters in Kazakhstan. Additionally, one oblast center in Kazakhstan had an article published in local a newspaper as a communication tool.

**Table 2:** The communication toolkit developed for Armenia (toolkits for other countries were similar but are not shown).

Target group	Communication materials
Veterinarians (small private vets and inspectors)	PowerPoints, posters, seminars, booklets
	Content: full range of what is needed for prevention and control of disease, including etiology

Farmers (small and large farmers)	Posters, booklets, meetings
	Content: introduction to African swine fever (ASF) and socioeconomic effect the disease can have

Hunters	Posters, booklets, meetings
	Content: the role of ticks in ASF transmission, and steps for limiting disease spread

For all groups	Agricultural TV channels: broadcast three TV programs
	Publication in magazines: three articles that cover issues about everything relevant for farmers (e.g., agriculture-based scientific magazine in Armenia)
	Content: how to report cases of disease

### Identification of Target Groups for Training Delivery

**Figure 2:**
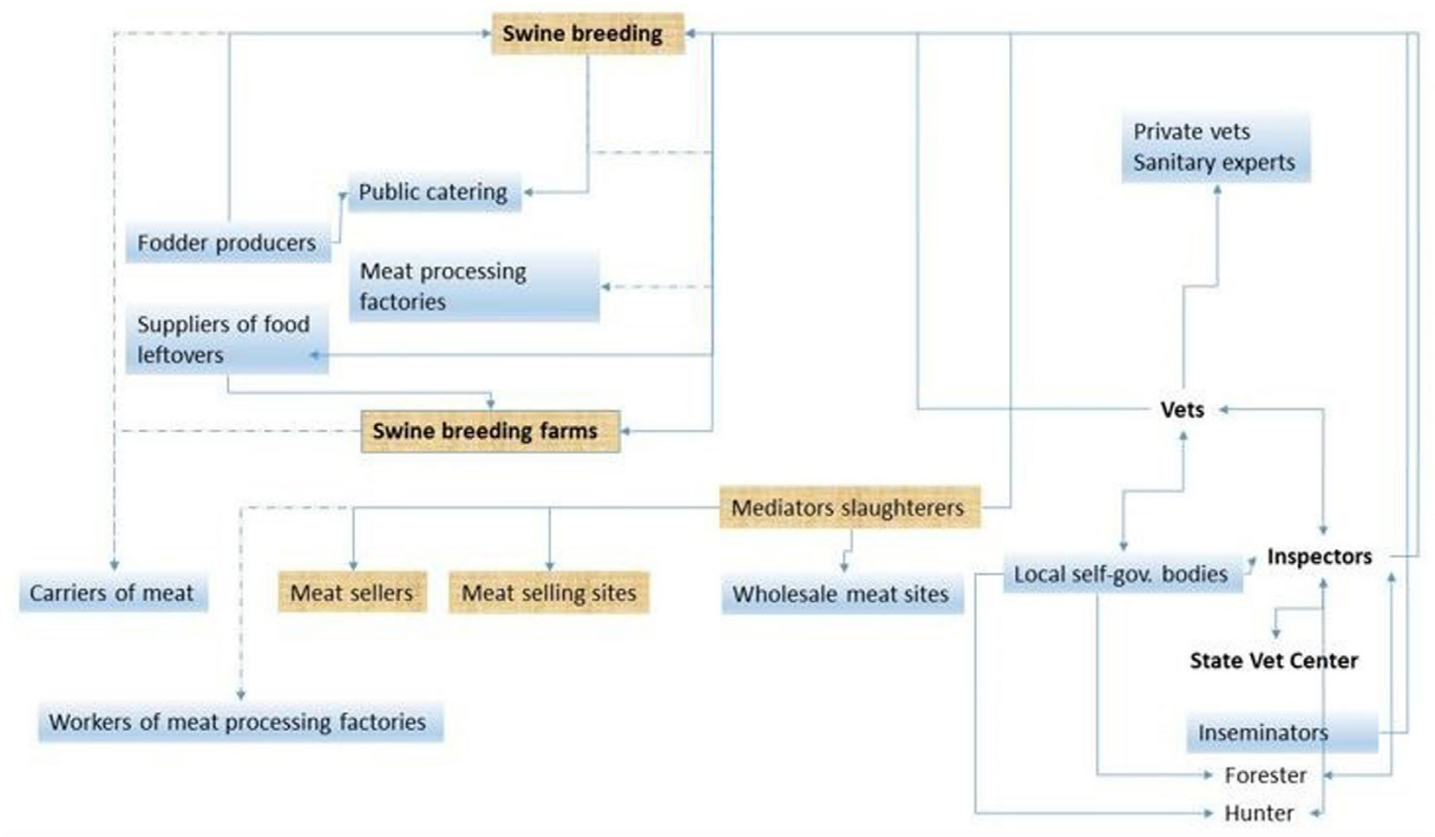
The stakeholder analysis diagram completed for Armenia (other countries’ data not shown). Each partner country developed a similar stakeholder analysis diagram identifying the principal constituents of the pig production chain. This analysis was used to identify principal target groups for each public outreach activity during the campaign.

To identify the target groups in each country, the principles of value chain analysis were introduced and applied during the second RM. The workshop participants were then asked to (i) describe the pig production chain and relevant stakeholders in their respective countries; (ii) identify final target groups for the outreach program; and (iii) finalize the objectives of the outreach program for each target group (Table 3). The target groups varied depending on an individual country’s needs and gap analyses. Participants from all four partner countries chose to target state and private veterinarians, farmers, and hunters for training; Kazakhstan’s delegates also chose to target meat processing plant managers. All four countries used the same process and analysis to identify principal target groups for each public outreach activity during the campaign. Armenia’s stakeholder analysis diagram is shown as an example in Figure 2. As part of the value chain analysis, principal stakeholders involved in the pig production chain in Armenia were identified. This formed the basis for selection of the target groups for outreach activities. The other countries representatives also used the same process and analysis to identify principal target groups for each public outreach activity during the campaign.

**Table 3:** The target groups and objectives that were developed for the outreach program in each of the partner countries.

Country	Target groups	Outreach program objectives
Armenia	Public Hunters Farmers State and private veterinarians	Increase general awareness about African swine fever (ASF)

Georgia	Famers State and private veterinarians	Show farmers how to apply ASF preventative measures Provide veterinarians with guidelines and SOPs for use in ASF cases

Kazakhstan	Farmers Hunters Veterinarians	Provide information about ASF risks and impacts Implement ASF information campaigns

Ukraine	Government Epi-zoologists Farmers	Disseminate ASF prevention information Provide information about ASF risks and impacts

*The target groups varied based on individual country needs and gaps as defined by the trainers in each country*.

### Strategies for Implementing the Outreach Program

Another working group session was focused on identifying the best strategies for implementing the outreach programs in each participating country. Country representatives in the working group session were asked to (i) identify realistic ways to integrate the goals of the project outreach program; (ii) determine which districts, provinces, and villages would be targeted in the outreach program; (iii) determine the number of participants that would be targeted by the outreach program; and (iv) discuss strategies to make the outreach program sustainable. In addition to developing implementation strategies for the outreach program, participants were also asked to develop a framework with which to assess the performance of the outreach programs. To do this, each country applied the logical framework concept during a country-specific working group session. Using this approach for each level of project implementation, country representatives identified objectives, indicators/targets, data sources, and personnel responsible for specific activities. For example, Kazakhstan defined the following tasks: conducting training for the trainers from each region in a ASF risk zone; developing a schedule for conducting workshops for in-country stakeholders, including veterinarians at the rayon level; providing population gathering in rayon and rural administrations for implementation of outreach goals on ASF with distribution of educational materials; and visiting farms to implement the ASF outreach program and distribute educational materials.

### Creating Implementation Plans for the Outreach Program

Once target groups were identified, partner countries created implementation plans for each of their outreach goals. The implementation plan included the implementation strategy (and possible integration with other ongoing activities in the field), the geographical coverage (number of districts and regions covered by the project), and other local stakeholders involved in outreach activities. In Armenia, outreach classes were conducted through marz-level meetings with community veterinarians. During these meetings, the representatives that were trained under the ToT program trained community veterinarians on core ASF topics and provided educational materials to distribute during outreach. Training delivery was assessed by project staff who had participated in the RMs.

### Faculty and Students

The main faculty of the project was SAFOSO, who served as the main facilitator and implementer, and the BTRICs and SMEs in each of the partner countries, who provided mentoring. The BTRICs and SMEs helped to foster relationships between the recipient countries and introduced various ASF and veterinary health-focused SMEs. Additionally, BTRICs assisted in the education of local nationals to develop and implement veterinary health awareness campaigns, including creating literature for target groups and organizations. Finally, BTRICs assisted local teams in preparing workshop agendas and delivering final schedules and workshops in their respective home countries.

Seventeen trainers were trained over the course of three RMs: five trainers from Armenia, four from Georgia, four from Kazakhstan, and four from Ukraine. A major goal of this program was training of trainers. These trainers were from the Ministries of Agriculture from each of their respective countries, and they became the “faculty,” or trainers, to the students (e.g., veterinarians, farmers, and hunters) in each country. These faculty trainers, after completion of the RMs, were equipped to conduct the public outreach campaign and student training in their home countries. The success of the program was then evaluated on the ability of these trainers to conduct the public outreach campaigns in their respective countries and disseminate the information on ASF that they developed.

## Metrics and Results To-Date

The trainers conducted training in their home countries after completing the training program.

- In Armenia, trainers conducted 10 regional events to train 20 regional trainers who ultimately trained veterinarians, farmers, and hunters.
- In Georgia, trainers conducted 16 regional trainings to train 100 district trainers and 377 private veterinarians.
- In Kazakhstan, four oblast-level trainings were conducted at oblasts bordering Russia, and 81 regional trainers were trained through 18 rayon training events.
- In Ukraine, one regional training event was conducted to train 14 regional trainers who then trained countless farmers and hunters.

The workshops and working group sessions established a foundation for the sustainability of ASF outreach programs and showed participants how to initiate future outreach programs. Each country could integrate the training program into their current educational program, thereby ensuring that the materials will continue to be presented to future cohorts.

### Soft Metrics

In a working group session, new trainers had the opportunity to receive feedback on their training delivery skills from the other trainers, BTRICs, SMEs, and SAFOSO. Country representatives evaluated presenter performance and the following aspects were discussed with the presenters: structure of the presentation, visualization, relevancy of content, timing, approach of the presenter, audiences’ understanding of the message, and technical language. Presentations and delivery methods were refined in response to this feedback.

### Evaluation of the Implementation of the Outreach Program

The effectiveness of this training was evaluated using the *Reaction* and *Learning* levels from Donald Kirkpatrick’s “The Four Levels of Learning Evaluation” (4). “The Four Levels of Learning Evaluation” is one of the most applied and accepted methods for evaluating training programs. *Reaction* assesses the trainees’ satisfaction with the trainers, and *Learning* examines the knowledge and skills gained during a training program. The two other levels from “The Four Levels of Learning Evaluation,” *Behavior* and *Results* require long-term evaluation and so were not assessed during this program.

### Assessment

The *Reaction* portion of the evaluation was designed to assess the trainees’ satisfaction with the training. Members of the working groups developed questionnaires for each country to be completed anonymously by trainees after each session. Participants chose different questions and scoring systems in their country-specific questionnaires but generally relied on either a four-point scale (excellent/good/fair/poor) or a binary scale (yes/no) to capture trainee impressions of the course, trainer, and program. The questionnaires also included space to write in additional comments. As an example, the 15 questions asked in the Georgian evaluation were:

- How interesting was the training?
- How understandable were the presentations?
- Did the presentation complete the objectives of the training program?
- How comprehensive was the information presented?
- Was there enough time for each presentation?
- Was the training relevant to work in Georgia?
- Did the trainer provide interactive engagement?
- How comprehensive was the trainer at answering your questions?
- Have the trainers brought appropriate practical learning examples?
- Were the trainers sociable or not?
- Were the presentations about relevant issues?
- Will the training materials be useful for you?
- How useful will the received knowledge be in your job?
- Was the training well-organized?
- Would you participate in further training?

### Learning

The *Learning* portion of the evaluation was designed to assess the knowledge and skills trainees gained during the in-country workshops. A pre-training test was administered at the beginning of the course to assess trainees’ baseline knowledge, and the same test (post-training test) was given at the end of the course to measure knowledge gained. Results from the pre- and post-training tests were compared to evaluate improvement in trainee understanding. Country representatives developed the pre- and post-tests during the RMs.

The questionnaires focused on six specific topics: etiology (one question) and epidemiology of ASF (10 questions), pathogenesis and immunology (three questions), clinical signs and pathology (four questions), sample management and laboratory diagnosis (three questions), and prevention and control (three questions). Only results from trainees who completed both a pre- and post-test were included in the analysis.

To assess the improvement of the trainees, we first calculated the median of percentages of correct answers by category of questions. Additionally, we calculated a global score (for pre- and post-tests) as the sum of right answers. This approach was also applied to specific categories or specific questions. We used the Wilcoxon signed-rank test for non-parametric data to evaluate the significance of improvement between pre- and post-tests.

## Implementation of the Training and Outreach Program

Planning for the ASF outreach program began during the first RM in Armenia in February 2015. Follow-up RMs took place in Kazakhstan, Ukraine, and Georgia later that year. Seventeen trainers developed the program and were effectively trained over the course of these RMs; these trainers went on to conduct in-country training and outreach activities between June and September 2015. As a result, 13,862 people were trained: 6,615 veterinarians, 7,110 farmers, 106 hunters, and 31 meat processing plant managers. In Armenia, there were 26 training events covering 10 regions. In Georgia, there were 117 trainings covering 11 regions and 61 districts. In Kazakhstan, there were 27 trainings covering 4 oblasts, 27 rayons, and 91 districts. In Ukraine, there were 14 trainings covering 14 oblasts. Table 4 summarizes the numbers of target groups reached in each country and the number of educational materials distributed through the public outreach campaign portion of this project.

**Table 4:** A summary of the target groups reached in each country and the numbers of educational materials distributed in the implementation of the public outreach campaign portion of this project.

Country		Target groups				Total
		Veterinarians	Farmers	Hunters	Others	
Armenia	Number of people reached by outreach program	301	2,000	100	N/A	2,401
	
	Educational materials distributed	1,500 booklets	1,500 booklets	150 posters	N/A	3,150

Georgia	Number of people reached by outreach program	100 (state vets)	108	N/A	N/A	585
		377 (private vets)				
	
	Educational materials distributed	497 guidelines	10,000 leaflets	N/A	N/A	10,497

Kazakhstan	Number of people reached by outreach program	76 (oblast level)	283 (large farmers)	6	31 (meat processing plant managers)	5,863
		748 (rayon level)	4,719 (small farmers)			
	
	Educational materials distributed	182 posters	7,769 leaflets	N/A	N/A	7,951

Ukraine	Number of people reached by outreach program	531 (Epi-zoologists district state administration and veterinary hospitals)	N/A	N/A	N/A	5,013
		4,482 (veterinaries at district hospitals)				
	
	Educational materials distributed	1,500 posters	100,000 leaflets	N/A	N/A	101,500

Total veterinarians reached						6,615

Total farmers reached						7,110

Total hunters reached						106

Total educational materials distributed						123,098

### Evaluation of the Training and Outreach Program by the Students

Trainer evaluations were conducted in each country to assess the utility of the outreach program. Table 5 shows the number of the respondents from Georgia. In total, 350 veterinarians from 11 regions completed a trainer evaluation form after training. Training was conducted in a classroom setting in each of the countries and the learning and outreach program was conducted in the form of workshops, lectures, and village level meetings. Trainees were asked to anonymously answer 15 questions about the trainer’s performance and the training program. Each aspect could be rated one (bad), two (medium), three (satisfactory), or four (good). At least 55% of the responses for each question were rated satisfactory or good, indicating positive reactions from the trainees; similar results were obtained in each partner country.

**Table 5:** Evaluation data from Georgia (data from other countries were similar but are not shown).

Region	Number of veterinarians who completed an evaluation form
Mtskheta Mtianeti	20
Shida Kartli	25
Kvemo Kartli	42
Samtskhe Javakheti	41
Kakheti	52
Imereti	69
Guria	19
Samegrelo	36
Racha	13
Adjara	27
Tbilisi	6
Total	350

*This is the overall data collected from the different regions in Georgia from the number of veterinarians who completed an evaluation form*.

**Figure 3:**
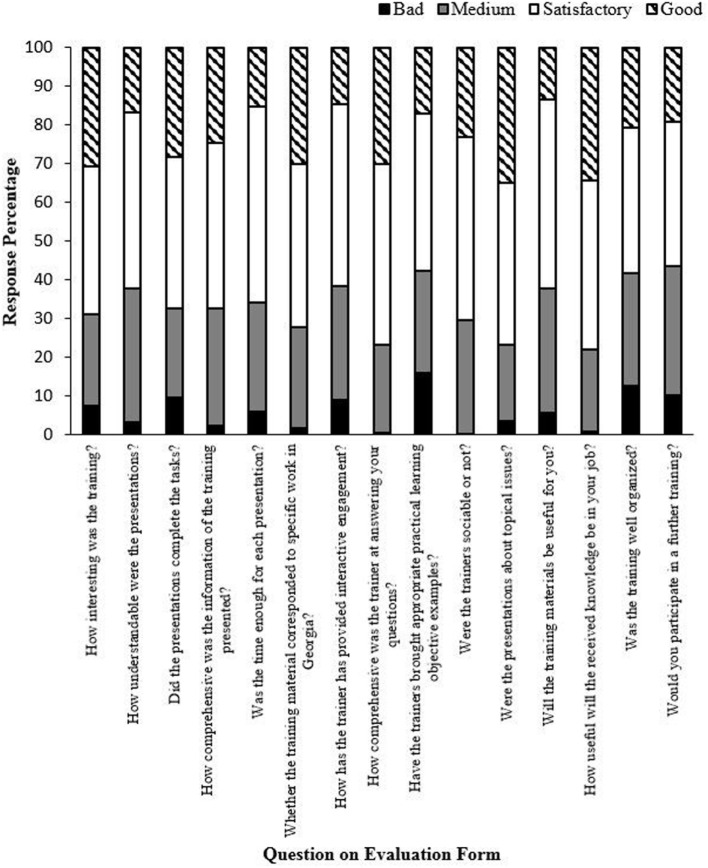
Trainee evaluation of the public outreach training program in Georgia (other countries’ data not shown). In Georgia, 350 veterinarians completed the evaluation form. Similar data were obtained from each partner country. The *y*-axis shows the percentage of each response for each question.

Figure 3 shows an example of trainees’ responses to the training program and trainers, as captured by the evaluation form developed for Georgia. Most Georgian trainees rated the program satisfactory or higher. In Kazakhstan, 392 veterinary workers, 20 farmers, and 6 hunting inspectors completed trainer evaluation forms. Approximately 50% of the responses for each question were rated as good, indicating positive reactions from the trainees. In Armenia, Georgia, and Kazakhstan, the average response rate to the *Learning* questionnaire was 75% (the total number of trainees who completed both a pre- and post-test). Individual country response rates were 95, 80, and 50% for Armenia, Georgia, and Kazakhstan, respectively. Results from Ukraine are not included because the pre- and post-tests were not completed by the same personnel and could no longer be obtained in this project because of an ongoing ASF outbreak.

**Figure 4:**
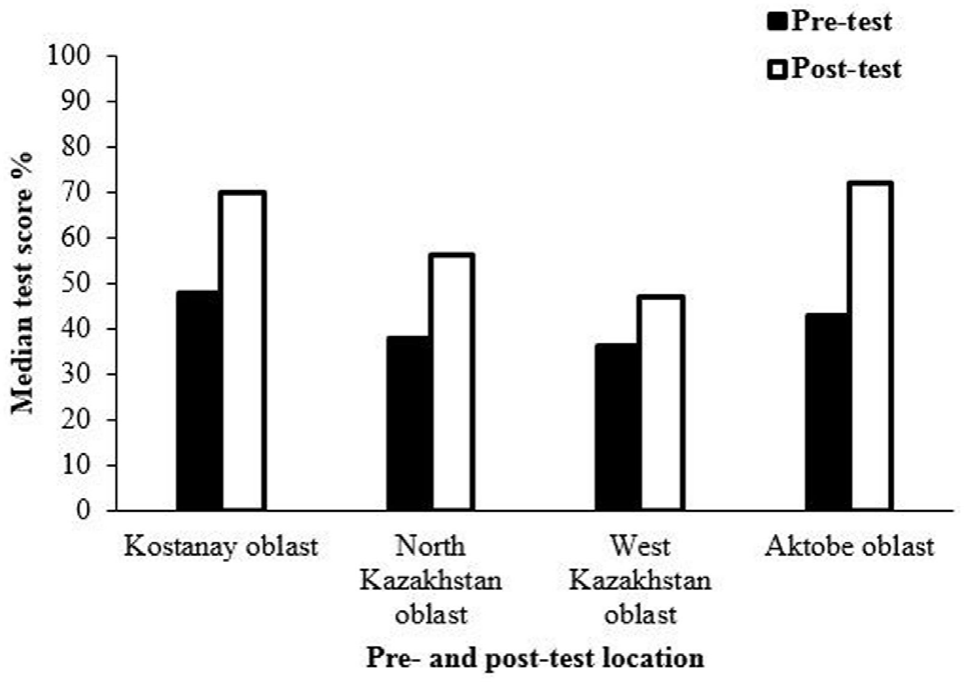
Median pre- and post-test results at the country level in Kazakhstan (other countries’ data not shown). Although the data did not indicate a significant improvement in trainee-specific knowledge after completing a training course in other countries, the training program exemplified the role of the training in a public outreach campaign. The questions used for evaluations varied from country to country, so improvements in trainees’ knowledge could not be reliably compared between countries.

Results shown in Table 6 are from in-country trainings that targeted trainers at the country level. In Georgia, the median percentage of right answers in post-training tests for each topic was consistently slightly higher than pre-training test scores. In Armenia, improvements in post-test scores varied depending on the knowledge category. In Kazakhstan, results were less satisfactory and are discussed in the Section “Discussion.” The differences between pre- and post-test scores were significant (*p* < 0.05) for Georgia and Kazakhstan according to the Wilcoxon test. Additionally, when comparisons are made between participants’ global scores (medians) in a country, participants’ knowledge improved slightly in Armenia and Georgia, with more evident improvement in Georgia. Only Georgia and Kazakhstan showed significant differences in global scores between pre- and post-tests (*p* < 0.05). Figure 4 shows the median pre- and post-test results at the country level in Kazakhstan. Although the data did not indicate a significant improvement in trainee-specific knowledge after completing a training course in other countries, the training program exemplified the role of the training in a public outreach campaign. In addition to the training evaluations conducted at the country level in Kazakhstan, pre- and post-tests were also completed at the oblast level. Trainees at the oblast level were trained by trainers who were trained and completed evaluations at the country level. Interestingly, trainees at the oblast level showed greater improvement after training than their trainers.

**Table 6:** Median pre- and post-test scores for trainings conducted at the central level in Armenia, Georgia, and Kazakhstan (data from Ukraine not shown), with 25–75% interquartile ranges.

Category	Armenia		Georgia		Kazakhstan	
	Pre-test% (Q1–Q3)	Post-test% (Q1–Q3)	Pre-test% (Q1–Q3)	Post-test% (Q1–Q3)	Pre-test% (Q1–Q3)	Post-test% (Q1–Q3)
Etiology of African swine fever (ASF)	50	47	70	93	40	93
Epidemiology of ASF	68 (54–78)	61 (46–71)	85 (77–95)	95 (90–95)	80 (49–88)	60 (43–60)
Pathogenesis and immunology	68 (63–79)	74 (68–82)	90 (88–93)	95 (95–96)	75 (63–78)	60 (60–60)
Clinical signs and pathology	87 (78–90)	90 (83–91)	78 (70–85)	93 (89–96)	80 (73–85)	60 (43–75)
Sample management and laboratory diagnosis	63 (55–75)	68 (55–75)	70 (63–80)	88 (86–93)	55 (33–60)	45 (38–55)
ASF prevention and control	90 (68–92)	90 (71–90)	80 (63–90)	95 (80–98)	90 (70–95)	70 (60–80)

## Discussion

As a result of this project, the governments of Armenia, Georgia, Kazakhstan, and Ukraine are equipped with a standard approach for developing outreach programs targeted at diseases of local concern. The ability to implement an effective public outreach campaign may help to reduce the future spread and incidence of diseases in these countries. Having a standard approach for implementing a public outreach campaign will allow the governments to rapidly disseminate important information to prevent or limit the spread of human, animal, and plant diseases in the future.

This program created a standard approach for conducting and applying a public outreach program to raise awareness of diseases of local concern. Implementation of the outreach program was achieved through training and development of an education campaign to enhance ASF public awareness by (i) identifying key personnel who could effectively implement necessary methods for reducing the spread of ASF; (ii) creating educational and training materials for dissemination to key personnel; (iii) educating key personnel on how to develop and implement a public awareness campaign; (iv) educating key personnel on the best strategies for mitigating the risk of ASF; and (v) developing a regional joint ASF working group.

The long-term success of the outreach program may ultimately be reflected in changes in the behavior and attitudes of the target groups as a result of their new knowledge of ASF spread and etiology. This may be manifested in improved swine health and, ultimately, the reduced spread and occurrence of ASF in the partner countries. The countries represented in this project are not the only ones with similar issues. A recent study of English pig farms showed that farmers have little knowledge about ASF clinical signs and are less concerned about ASF than other pig diseases (5). The pig farmers in the study lacked awareness of outbreaks in other countries and believed that reporting ASF would have a negative impact. These findings suggest important areas for educational campaigns targeted to English pig farms to focus on increasing the likelihood responding to an ASF outbreak. The project and implementation we described in this paper could be used in other countries to develop similar programs. Continuing program success will be measured by the number of swine disease cases, specifically the number of ASF outbreaks. Regional and state veterinarians, and government agencies in Armenia, Georgia, Kazakhstan, and Ukraine will be responsible for measuring long-term success through analysis of ASF lab results, monthly disease reports, and messages from veterinarians. It will take several years to generate enough data to determine if the outreach program succeeded in mitigating ASF disease outbreaks.

The success of the training and outreach program is reflected in the overall increase in trainee knowledge about the disease, the large population that was reached, the number of educational materials that were distributed, and trainees’ satisfaction with the outreach and training program. Trainees in the program showed improvement in ASF knowledge after completing a training course, based on the results of pre- and post-test evaluations. In some cases, results were less satisfactory than expected. Trainers facilitated follow-up discussions on the results of the surveys immediately after the trainings, and participants highlighted some limitations of the approach.

The outreach classes reached over 13,000 farmers, veterinarians, and hunters in Armenia, Georgia, Kazakhstan, and Ukraine. Partner country representatives received training on how to establish different technical and didactical capacities to successfully engage the target groups. Although many people participated, it was uniformly difficult to engage hunters and backyard farmers in the partner countries. Engaging hunters in the ASF outreach program is especially important, due to the need of a system where wild boar carcasses are regularly reported. Future campaigns should identify ways to reach more hunters, such as by engaging hunters’ associations. Kazakhstan has discussed the need to develop a network between veterinarians and small farmers, hunting entities, and managers of the meat industry.

Although the program was tailored to each target group, it was not entirely successful. Georgian representatives noted that farmers were generally distrustful of veterinarian authorities and were often reluctant to follow recommendations from them. Additionally, backyard farming is usually a secondary household income, so there are few incentives and financial resources to improve biosecurity. Bringing about a change in attitude is not an easy task and will require long-term efforts. Future awareness campaigns should consider the social structure and network of the target groups to develop the most effective strategies for reaching them. For example, hunters might be better engaged by inviting members of hunters’ associations to participate in official meetings and workshops. Educational materials need to be continually renewed and improved for greater relevance to the target groups. Finally, to increase the impact of future projects, entities with a strong network at the village/community level (e.g., non-governmental organizations, research institutes, and private companies) should be involved in future disease awareness campaigns alongside veterinarians.

## Conclusion

This project has provided each participating country with a sustainable public outreach program designed to educate local agricultural workers and first responders about ASF. Collectively, the effective implementation of these outreach programs has increased regional communication on the status of ASF and enhanced public knowledge. In addition, the approach used by this project was the first such effort that facilitated the development of the capacity to implement outreach campaigns for future disease outbreaks in the region. During this project, recipient countries were taught how to run an outreach campaign, including how to identify target audiences, and how to produce effective educational materials. This project also helped to establish and encourage collaboration between members of international veterinary communities. Because of this project, each partner country now has the tools to carry out future disease awareness campaigns. This project helped establish regional cooperation amongst multiple countries involved in preventing the spread of ASF.

## Author Contributions

MN led and conducted the training program as a consultant, acted as a project mentor and implementer, and assisted with the writing of the manuscript. AL completed the statistical analysis of the test surveys. The following authors contributed to the implementation of the program and training and were part of the initial writing of the manuscript: TS, BK, TK, IS, ST, AA, SN, MS, ON, RD, TC, LA, OP, LN, NK, TN, ZA, DK, IM, and MZ. The following authors assisted as an implementer in each country and assisted with in-country coordination: LN, NM, BB, GZ, SS, MM, TG, and RO. TG designed the initial concept of the project, created the overall design, and structured the regional training approach and assisted with writing the proposal, reporting to the funding agency, and, implementation plan, and manuscript. RO served as the project manager, and assisted with the concept design, manuscript writing, and overall project implementation and manuscript writing.

## Conflict of Interest Statement

MN is employed by SAFOSO and was a project mentor and implementer. AL is employed by SAFOSO and worked on statistical analysis. TG and RO are employed by Avila Scientific. All authors declare no competing interests. The authors declare that the research was conducted in the absence of any commercial or financial relationships that could be construed as a potential conflict of interest.

## References

[1] Galindo I Alonso C African swine fever virus: a review. Viruses (2017) 9(5):103 10.3390/v9050103 PMC5454416 28489063

[2] Food and Agriculture Organization of the United Nations/World Organization for Animal Health/World Bank. Good Practices for Biosecurity in the Pig Sector – Issues and Options in Developing and Transition Countries. FAO Animal Production and Health Paper No. 169. Rome: FAO (2010). Available from: http://www.fao.org/3/a-i1435e.pdf

[3] Rowlands RJ Michaud V Heath L Hutchings G Oura C Vosloo W African swine fever virus isolate, Georgia, 2007. Emerg Infect Dis (2008) 14(12):1870–4. 10.3201/eid1412.080591 19046509 PMC2634662

[4] Kirkpatrick D Kirkpatrick J Evaluating Training Programs: The Four Levels. San Francisco, CA: Berrett Koehler Publishers (2006).

[5] Guinat C Wall B Dixon L Pfeiffer D English pig farmers’ knowledge and behavior towards African swine fever suspicion and reporting. PLoS One (2016) 11(9):1–13. 10.1371/journal.pone.0161431 PMC5042443 27684556

